# Establishing a Standardized DNA Extraction Method Using NaCl from Oral Mucosa Cells for Its Application in Imprinting Diseases Such as Prader–Willi and Angelman Syndromes: A Preliminary Investigation

**DOI:** 10.3390/genes15050641

**Published:** 2024-05-18

**Authors:** Letícia Lopes Cabral Guimarães da Fonseca, Danielle Nascimento Rocha, Hiago Azevedo Cintra, Luiza Loureiro de Araújo, Gabrielle Leal Monteiro dos Santos, Leonardo Lima de Faria, Margarida dos Santos Salú, Silvia Helena dos Santos Leite, Adriana Duarte Rocha, Maria da Conceição Borges Lopes, Igor Ribeiro Ferreira, Leonardo Henrique Ferreira Gomes, Letícia Cunha Guida

**Affiliations:** 1 Instituto Nacional da Saúde da Mulher, da Criança e do Adolescente Fernandes Figueira–Fundação Oswaldo Cruz, Rio de Janeiro 22250-020, Brazilhiagoazevedo97@gmail.com (H.A.C.); luiza.loureiro@fiocruz.br (L.L.d.A.); leo.nardolimadefaria@gmail.com (L.L.d.F.);; 2 Rural and Remote Support Services, Department of Health, Integrated Cardiovascular Clinical Network SA, Adelaide 5042, Australia

**Keywords:** oral swab, Prader–Willi syndrome, molecular diagnostics, imprinting disorder

## Abstract

Background: Diagnosing imprinting defects in neonates and young children presents challenges, often necessitating molecular analysis for a conclusive diagnosis. The isolation of genetic material from oral swabs becomes crucial, especially in settings where blood sample collection is impractical or for vulnerable populations like newborns, who possess limited blood volumes and are often too fragile for invasive procedures. Oral swab samples emerge as an excellent source of DNA, effectively overcoming obstacles associated with rare diseases. Methods: In our study, we specifically addressed the determination of the quality and quantity of DNA extracted from oral swab samples using NaCl procedures. Results: We compared these results with extractions performed using a commercial kit. Subsequently, the obtained material underwent MS–HRM analysis for loci associated with imprinting diseases such as Prader–Willi and Angelman syndromes. Conclusions: Our study emphasizes the significance of oral swab samples as a reliable source for obtaining DNA for MS–HRM analysis. NaCl extraction stands out as a practical and cost-effective method for genetic studies, contributing to a molecular diagnosis that proves particularly beneficial for patients facing delays in characterization, ultimately influencing their treatment.

## 1. Introduction

Obtaining high-quality DNA marks the initial step in molecular diagnosis, particularly when subsequent procedures might compromise DNA quality—such as bisulfite conversion in methylation pattern studies. While peripheral blood is conventionally used, challenges arise in collecting samples from children and shipping materials from patients and family members residing in remote and inaccessible locations. In this context, collecting material through oral mucosa cells, commonly employed in forensic medicine, emerges as a viable alternative for DNA extraction [1,2,3].

Buccal swabs are painless, noninvasive, and simple to collect and have been used to detect genetic material (DNA and RNA) and pathogen-specific antibodies in various viral infections, including dengue, hepatitis B, measles, rubella, and parvovirus B19 [4,5,6,7].

The use of oral swabs in forensic medicine is well-documented in the scientific literature, with several studies highlighting their crucial applications. For instance, a study by Raposo et al. (2019) examined the use of oral swabs in collecting DNA samples in cases of sexual assault, demonstrating their effectiveness in obtaining genetic material from the aggressor in the victim’s oral cavity [8]. Additionally, research such as that of Silva et al. (2017) explored the use of oral swabs in identifying cadavers, showing that these devices allow the collection of genetic material for DNA analysis when blood samples are not available or inadequate [9]. In criminal investigations, studies like that of Gomes et al. (2020) investigated saliva sample collection for DNA analysis using oral swabs, highlighting their importance in obtaining crucial evidence for case resolution [10]. In summary, the scientific literature confirms that oral swabs are indispensable tools in forensic medicine, playing a fundamental role in evidence collection and individual identification in various investigative scenarios.

In addition to the mentioned applications, oral swabs are widely used in forensic genotyping, providing valuable information for individual identification and case resolution. Studies such as that of Smith et al. (2018) investigated the efficacy of oral swabs in genotyping genetic markers, demonstrating their utility in obtaining accurate genetic profiles from saliva samples [11]. These findings are supported by research such as that of Jones et al. (2020), which highlighted the importance of oral swabs in DNA genotyping for identifying suspects and victims in criminal investigations [12]. Therefore, besides their applications in DNA sample collection, oral swabs also play a crucial role in forensic genotyping, significantly contributing to case resolution in judicial proceedings.

Rare diseases are chronic and disabling disorders that affect 65 or fewer per 100,000 individuals, according to the Ministry of Health of Brazil, and 80% of these cases have been described with a genetic etiology [13,14]. Genomic imprinting is a phenomenon that results in the parent-specific expression of a small number of genes. Imprinted genes are usually found in clusters and regulated by imprinting control regions, which exhibit parent-specific DNA methylation acquired during germline development. Comprehensive analysis of DNA methylation patterns is critical for understanding the molecular basis of many human diseases [15]. Clinical examination alone hinders the diagnosis of imprinting defects in newborns and children. The clinical and laboratory diagnosis of rare diseases associated with imprinting defects is a complex endeavor that demands the amalgamation of various molecular biology and cytogenetic techniques to elucidate the mechanisms underlying disease development [16]. Currently, the use of “omics” sciences such as NGS sequencing is being considered, but the costs are still prohibitive when considering screening strategies, and they require infrastructure for analysis.

At the screening stage, DNA methylation analysis must be able to distinguish the few patients from the vast majority of laboratory references that fall into the differential diagnosis at birth and later make the test as easy to use as possible—it is challenging to obtain blood or other patient tissue, and family availability is not always guaranteed; also, consideration must be given to the impact of the test cost on the public health system [16].

Genomic imprinting loss has been identified as the cause of several human syndromes (reviewed in Biliya and Bulla, 2010). The most well-characterized syndromes are Angelman syndrome (AS; OMIM #105830), Prader–Willi syndrome (PWS; OMIM #176270), Beckwith–Wiedemann syndrome (BWS; OMIM #130650), Silver–Russell syndrome (SRS; OMIM #180860), transient neonatal diabetes mellitus type 1 (TNDM; OMIM #601410), pseudohypoparathyroidism type 1A (PHP1A; OMIM #103580) and type 1B (PHP1B; OMIM #603233), Temple or Maternal uniparental disomy of chromosome 14 syndrome (TS or UPD(14)mat; OMIM #616222), and Kagami–Ogata or Paternal uniparental disomy of chromosome 14 syndrome (KOS or UPD(14)pat; OMIM #608149) [17].

The PWS is a genetically complex disorder resulting from abnormalities in the 15q11-q13 region of the paternal allele of chromosome 15. The clinical diagnosis of PWS is challenging due to phenotypic overlaps with other genetic diseases, such as Schaaf-Yang Syndrome (SYS) and various Prader–Willi-Like (PWLS) clinical picture presentations. Shared phenotypic features include neonatal hypotonia, feeding difficulties, weight gain, intellectual developmental delay, and obesity [18,19,20,21]. Laboratory characterization of PWS through molecular techniques (MS–HRM, MS-MLPA, and Microsatellite Analysis) and molecular cytogenetics (FISH) distinguishes it from other genetic syndromes, shedding light on the genetic mechanisms underlying the syndrome [22]. However, the current diagnostic process is laborious, time-consuming, and costly due to the need for multiple techniques. The methylation pattern analysis of exon 1 of the *SNURF-SNRPN* gene using the MS–HRM technique effectively detects 99% of PWS cases. Although it cannot differentiate between the genetic mechanisms causing the syndrome, its simplicity, low cost, and ease of result interpretation make it an excellent screening method [23,24].

Ensuring an early genetic diagnosis is paramount, as it averts unnecessary interventions and facilitates prescribing appropriate treatments. Nevertheless, obstacles persist in the conventional method of peripheral blood collection, especially when dealing with pediatric patients or those residing in remote and challenging-to-access areas [13]. Some patients face limitations due to their reduced blood volume, making invasive procedures complex, and obtaining parental consent, particularly in the case of neonates, can prove challenging [13,14].

Utilizing oral cells as a material source for DNA extraction presents a promising alternative [23]. This user-friendly collection method allows parents to quickly obtain an oral mucosa sample using a provided kit with clear instructions for buccal swab collection. Subsequently, they can dispatch the sample to a local molecular laboratory for confirmation tests [24]. Consequently, a pressing need exists to standardize noninvasive biological sources for diagnostic purposes.

In this study, we utilized the NaCl DNA extraction technique on oral mucosa cells to assess the quality of the obtained DNA for subsequent conversion steps and Methylation-Sensitive High-Resolution Melting (MS–HRM) analysis, serving as a model for studying the Imprinting Center of PWS and AS. The standardization of this DNA extraction method from oral mucosa cells using NaCl, a process previously established for peripheral blood DNA extraction [25], was conducted and compared with a commercial kit. The assessment of DNA quality involved the analysis of the methylation profile of the imprinting center located in the promoter region of exon1 of the *SNURF-SNRPN* gene. This region, linked to Prader–Willi and Angelman syndromes, was a model for patients suspected of imprinting defects.

## 2. Materials and Methods

### 2.1. Sample Collection

In this study, two oral swabs were collected randomly from mothers enrolled during our regular prenatal follow-up from each of the 90 babies born at Fernandes Figueira Institute (IFF). No selection criteria were used for our sample, which should reflect the general population. In addition to the baby swabs, the study included ten individuals (aged 6–18 years) with a previous diagnosis of PWS confirmed by the MS-MLPA technique and another ten individuals with clinical suspicion of PWS. In addition to the 20 oral swab samples, 20 fresh peripheral blood samples were collected (10 from patients suspected of having PWS and 10 samples of fresh blood from individuals with confirmed diagnoses) and used as controls. The suspected individuals ranged in age from months to 10 years and exhibited a combination of phenotypes such as severe hypotonia, poor appetite, and feeding difficulties in early infancy, followed in early childhood by excessive eating and gradual development of morbid obesity. Motor and language development milestones were delayed [26].

The study was conducted by the Declaration of Helsinki and approved by the Fernandes Figueira Institute IRB (CAAE: 45767015.0.0000.5269). The Informed Consent Statement was obtained from all guardians of subjects involved in the study. Written informed consent was obtained from the participants’ guardians to publish this document.

### 2.2. DNA Extraction Protocols

The procedure followed that described by Abrão et al. [27]; oral cotton swabs were collected from newborns and patients. Patients were instructed to rinse with distilled water, and the collection was performed by scraping the inner face of the cheeks with small sterile cytological brushes and making circular movements approximately 10–20 times. The brushes had the external portion of the stems cut and placed in microtubes.

The extractions were performed immediately or after refrigeration (4–6 °C) for 2 to 30 days before extraction.

Genomic DNA isolation from oral swabs was performed using two different protocols:

The method with NaCl: In the NaCl extraction, 200 µL of TES (10 mM Tris HCl pH 7.6; 1 mM EDTA; 0.6% SDS) and 5 µL of proteinase K (10 mg/mL) were added to the tubes containing the swab and incubated for 2 h at 42 °C. After incubation, the brush was pressed against the wall of the tubes and removed. A final volume of approximately 250 µL was obtained, to which 42 µL of saturated NaCl [26] was added, shaking vigorously by hand centrifuge for 1 min at 15,000× *g*. The supernatant was transferred to a new tube, and two times the volume of absolute ethanol was added. The tubes were shaken and centrifuged for 1 min at 15,000× *g*. Absolute ethanol was discarded, and 1 mL of 70% ethanol was added, inverting the tubes several times to wash the pellet. The tubes were centrifuged for 1 min at 15,000× *g*, and the supernatant was discarded. Washing with 70% ethanol was repeated one more time, and after discarding the supernatant, the tubes remained open for 30 min to evaporate the residual ethanol, as described by Abrão et al. [16]. The DNA was dissolved in 25 µL of 1X TE (10 mM Tris HCl; 0.1 mM EDTA).

Extraction with the Commercial Kit Qiagen-DBS: DNA isolation was performed with the QIAamp DNA Mini kit (QIAGEN, Germantown, MD, USA) following the manufacturer’s protocol with AL buffer. The same DNA extraction method was performed with the peripheral blood from Prader–Willi, Angelman, and healthy control patients.

DNAs were stored at 4 °C, and subsequent steps (conversion and MS–HRM) were conducted within three months after extraction.

### 2.3. DNA Quantification

DNA concentration (ng/μL) was determined using a Qubit 2.0^TM^ Fluorometer with dsDNA BR Assay Kit^TM^ for Qubit and a NanoDrop™ 2000 Spectrophotometer (Thermo Fisher Scientific, Waltham, MA, USA). The DNA purity (260/280 and 260/230 ratios) was assessed by NanoDrop 2000 Spectrophotometer (Thermo Fisher Scientific, Waltham, MA, USA). The two extraction protocols determined the concentration and purity from swabs and peripheral whole blood samples.

### 2.4. DNA Integrity

The integrity of genomic DNA was tested by resolving DNA extracts on a 0.8% agarose gel by electrophoresis (Bio-Rad, Hercules, CA, USA), followed by visualization with ethidium bromide staining. Each DNA sample was graded according to the electrophoretic migration of sample DNA compared with a known molecular weight marker (λDNA/Hind III fragments, Invitrogen, Carlsbad, CA, USA).

### 2.5. Ribonuclease P (RPP38) Amplification

To ensure DNA integrity and to exclude the possibility of false negatives due to the presence of eventual inhibitors, the TaqMan RPP38 Control Reagents kit (Catalog number 4316844, Applied Biosystems, Foster City, CA, USA) was used as a reference amplification control following the manufacturer’s protocol. All reactions were performed in a MicroAmp Fast Optical 96-Well Reaction Plate using the 7.500 Fast Real-Time PCR System Mix (Applied Biosystems, Foster City, CA, USA).

### 2.6. Bisulfite Treatment

A total volume of 20 μL [20 ng/μL] of DNA extracted from swabs and peripheral blood was treated with an EZ-96 DNA Methylation-Gold Kit (Zymo Research, Irvine, CA, USA), following the manufacturer’s protocol. Bisulfite-converted DNA was quantified by NanoDrop 2000 Spectrophotometer (ThermoFisher Scientific, Waltham, MA, USA).

Once converted, the DNA was subjected to MS–HRM analysis within 24 h.

### 2.7. Methylation-Sensitive High-Resolution Melting (MS–HRM)

The MS–HRM was performed in triplicates with the bisulfite-treated DNA isolated from each individual’s swabs or whole peripheral blood. It was performed in a MicroAmp Fast Optical 96-Well Reaction Plate using the 7500 Fast Real-Time PCR System Mix (Applied Biosystems) with the primers 5′-GGATTTTTGTATTGCGGTAAATAAG-3′ and 5′-CAACTAACCTTACCCACT CCATC-3′ (forward and reverse, respectively) as previously described by Ferreira et al. [25,28] (Additional File S1). These primers hybridize to the imprinting center in the promoter region of exon1 of the *SNURF/SNRPN* gene associated with Prader–Willi and Angelman syndromes. The melting temperatures of 78.8 °C and 83.3 °C were chosen as a near-proportional amplification of unmethylated and methylated alleles, respectively.

### 2.8. Statistical Analysis

Each group analysis was carried out with the unpaired Student’s *t*-test to detect differences. A two-sided *p*-value < 0.05 was considered statistically significant. The *p*-value expresses a degree of confidence. If the probability of this result occurring is minimal, we can conclude that the observed result is statistically relevant. This probability is also called *p*-value or *p*-value. Consequently, the level of confidence *α* is equal to 1—*p*-value. Percentile, mean, median, and standard deviation values of RPP38 amplifications were also calculated for comparative purposes.

## 3. Results and Discussion

This study presents a rapid, economical, and robust method for obtaining genomic DNA from human oral swabs. It requires minimal sample volume but achieves optimal concentration and purity for qPCR and MS–HRM for methylation profile analyses.

Peripheral blood samples were included as controls in the study. Nevertheless, the notable disparity in sample numbers between peripheral blood (20 tubes) and oral swabs (2 × 110) introduced limitations in conducting statistical comparisons. Consequently, our analysis primarily examined the outcomes derived from material extracted from oral swabs utilizing the two distinct extraction methods.

**Figure 1:**
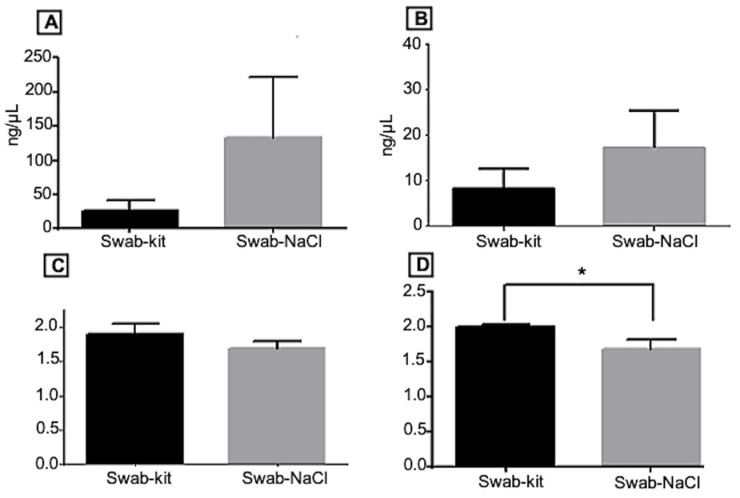
Comparison between DNA extraction methods (commercial kit and NaCl) from oral swabs. Legend: In (**A**,**B**), the average DNA concentration was measured with NanoDrop (**A**) and Qubit (**B**). In (**C**,**D**), the Purity parameters for 260/230 and 260/280, respectively, are shown. Swabs extracted with a commercial kit are shown in black rectangles, and swabs extracted with NaCl are shown in gray rectangles. * means *p*-value < 0.05 and considered statistically significant.

The quality and quantity of the DNA samples extracted from the oral swabs and a DNA sample are crucial for the analyses that followed in this work. The DNA concentration was higher with the NaCl methodology (131.38 ng +/− 85.54 in NanoDrop and 17.21 ng +/− 10.84 in Qubit) compared to Qiagen (26.12 ng +/− 14.13 in NanoDrop and 8.60 ng +/− 4.88 in Qubit); these differences proved inconsequential due to the substantial variability in DNA amounts obtained through the NaCl extraction method (Figure 1A and Supplementary Materials S2).

Analysis of purity parameters (260/280 and 260/230) revealed lower NaCl values than the commercial kit (refer to Figure 1B,C and Supplementary Materials S2). Concerning the 260/280 wavelength ratio, the differences were 1.91 (+/− 0.14) for the commercial kit and 1.69 +/− 0.11 for NaCl extraction and the 260/230 nm ratio, with values of 1.99 (+/− 0.03) for the kit and 1.68 (+/− 0.13) for NaCl extraction. These significant differences in the 230 nm ratio can be explained, for this difference could be the residual presence of SDS (a detergent commonly used in the cell lysis step) in the initial stages of DNA extraction, even when employing commercial kits or home methods [29]. Despite this variation, no issues were detected in subsequent steps, including real-time amplification, bisulfite conversion, and dissociation curve analysis.

In this study, samples of purified DNA obtained from oral swabs using NaCl and commercial kits and from blood were subjected to agarose gel electrophoresis (0.8%). Since larger genomic DNA molecules migrate more slowly than smaller DNA and RNA fragments, visualization of the agarose gel revealed distinctive patterns. Specifically, NaCl-extracted samples exhibited a pronounced trailing effect, and the bands were predominantly located at the gel’s lower end, indicating potential contamination from small RNA molecules or degraded DNA fragments. This observation suggests that the quantification of genomic DNA extracted via NaCl may be inflated due to the concurrent presence of smaller nucleic acid molecules, therefore influencing absorption measurements at 260 nm.

These findings offer insights into the disparities between quantification methods such as NanoDrop and Qubit, particularly in instances involving the presence of degraded RNA and DNA molecules. Notably, the fluorophore employed in the Qubit system specifically targets DNA, potentially explaining the observed differences. Despite the heightened intensity of the trailing effect and lower bands, bands larger than 12,000 bp were still discernible, as demonstrated by the data provided in Additional File S3.

**Figure 2:**
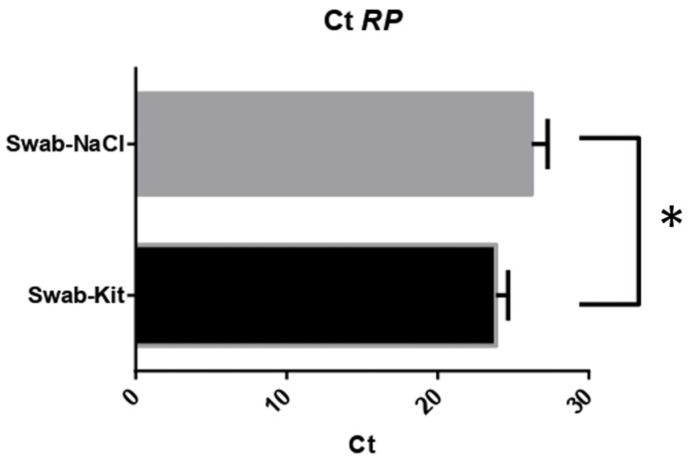
Graph comparing the means of amplification Ct for the *RNAse P* gene with DNA extraction methods. * means *p*-value < 0.05 and considered statistically significant.

A real-time qPCR was performed for the *RNAse P* gene for all samples extracted to evaluate the efficiency of extractions of genetic materials and the presence of inhibitors. The DNA of the extracted samples was directly subjected to these qPCRs. Nevertheless, this degradation pattern does not appear to disrupt the qPCR reaction. The subsequent step involved amplifying target regions through the real-time PCR technique. Initially, the tested region was the human P/MRP ribonuclease gene *RPP38* subunit; all samples were amplified irrespective of the extraction protocol. The average Ct was 23.87 (+/− 0.78) for samples extracted with the commercial kit and 26.87 (+/− 1.45) for those extracted with NaCl. This primer pair for the specified region exhibited a significant difference in the detection cycle (Ct) when comparing the two protocols (Figure 2 and Additional File S1).

**Figure 3:**
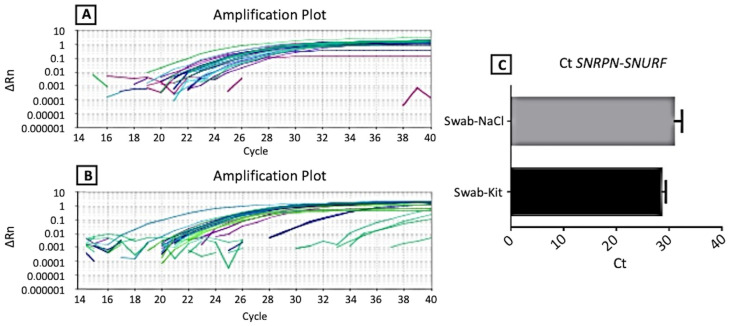
Amplification curves of the exon 1 promoter region gene of the *SNRPN-SNURF* genes in the imprinting center (IC) region with the DNAs extracted by the two methods. Legend: In (**A**), the amplification curve with the material extracted with the Commercial Kit; in (**B**), the amplification curve with the material extracted with NaCl; and in (**C**), graphs of comparison between the means of amplification Ct for the *SNRPN-SNURF* gene compared to DNA extraction methods. Swabs extracted with a commercial kit are shown in black rectangles, and swabs extracted with NaCl are shown in gray rectangles.

The analysis of the amplification of the promoter region of exon 1 of the *SNRPN-SNURF* genes in the imprinting center (IC) region revealed an average Ct of 28.59 (+/− 0.80) for samples extracted with the commercial kit and an average Ct of 30.88 (+/− 1.48) for samples extracted with NaCl. It is crucial to note that, unlike the observations for the *RNAse P* gene, these differences were not statistically significant (Figure 3 and Supplementary Materials S2).

**Figure 4:**
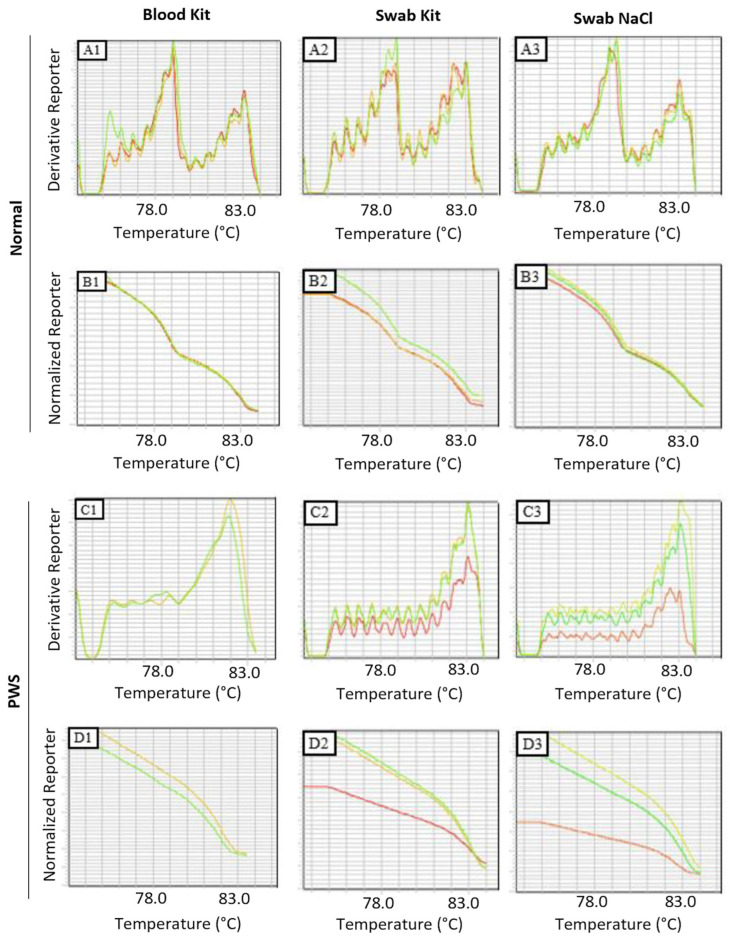
Methylation pattern analyzed by MS–HRM. Legend: The dissociation curves show the methylation peak for each allele. Normal patients, in the dissociation-derived curves (**A1**–**A3**), present two peaks corresponding to unmethylated paternal alleles and methylated maternal alleles; (**A1**) is the result of the technique performed using the kit from peripheral blood, (**A2**) with the use of the kit from oral swab and (**A3**) performed by NaCl from an oral swab. The graphs of the normalized curves show the normal patients present fluorescence corresponding to the paternal and maternal alleles and two sharp drops (**B1**–**B3**). The absence of the paternal allele was detected due to the presence of a single peak in the derived curve (**C1**–**C3**) and a single drop in the derived curve (**D1**–**D3**), confirming the diagnosis of Prader–Willi. The analyses seen in (**C1**,**D1**) were performed from peripheral blood, (**C2**,**D2**) using the kit from an oral swab, and (**C3**,**D3**) by NaCl from an oral swab. The methylation temperature detected for the unmethylated paternal allele was close to 78.8 °C in all cases, and for the methylated maternal allele, it was around 83.3 °C in all cases. The different colours means experimental triplicates.

Converting the bisulfite-extracted DNA transformed unmethylated cytosines in the paternal allele into uracil, leaving the methylated cytosines in the maternal allele unchanged. The converted DNA was subjected to MS–HRM analysis to examine the methylation patterns of paternal and maternal alleles of the *SNRPN-SNURF* gene (Figure 4). Furthermore, MS–HRM was performed on 180 swab samples from the population without clinical suspicion, from ten individuals with a previous diagnosis of PWS confirmed by the MS-MLPA technique, and from another ten individuals with clinical suspicion of PWS. In swabs collected from the general population born at IFF, the results were concordant in both extraction types and showed a normal methylation pattern. MS–HRM results from swabs and peripheral blood samples were concordant in individuals previously identified by MS-MLPA and those with clinical suspicion. Among the ten individuals identified by MS-MLPA, their diagnoses were confirmed in peripheral blood samples and DNA extracted from swabs.

Furthermore, individuals with clinical suspicion showed no disagreement between the results obtained from peripheral blood and oral swab samples. Among the ten suspected individuals, four were considered normal for PWS and AS, as indicated by a methylation pattern presenting two peaks related to dissociation temperature—the first peak (78 °C) corresponding to the paternal (unmethylated) allele and the second peak (83 °C) to the maternal (methylated) allele (Figure 4A). The other six patients presented a methylation pattern associated with Prader–Willi syndrome. The PWS pattern is characterized by a single peak related to the dissociation temperature of the maternal allele and the absence of the peak associated with the paternal allele (Figure 4C). Samples collected from newborns consistently displayed a methylation pattern in peripheral blood and oral swab material.

Examination of the normalized curves revealed minimal differences in profile between patients with standard methylation profiles (Figure 4B1–B3) and those with profiles consistent with PWS (Figure 4D1–D3). The derived curve demonstrated a slight distinction in the maternal allele in NaCl-extracted swab samples compared to commercial kit-extracted samples and peripheral blood in individuals with standard methylation patterns (Figure 4A1–A3). However, the normalized curves exhibited similar behavior in individuals with a methylation pattern indicative of PWS (Figure 4C1–C3). A significant emphasis is that despite a lower degree of purity in the NaCl method versus commercial kit, the diagnostic results (PWS vs. non-PWS) remained consistent.

Efficient drug intervention is directly linked to a rapid and accurate diagnosis. For instance, Growth Hormone (GH) administration in PWS patients has demonstrated various benefits, with optimal effects observed when initiated in the first few years of life. However, the lengthy diagnostic process, requiring specialized technical teams, may compromise the timing of GH treatment [30].

The overlap of phenotypes poses a diagnostic challenge, especially in newborns. One example is the cases of hypotonia present in various conditions, such as metabolic diseases, acute or chronic illnesses, genetic syndromes, endocrinopathies, myopathies, and abnormalities of the central or peripheral nervous system [31]. While our understanding of the genetic basis of some clinical phenotypes has advanced significantly in the last decade [32,33,34], there is still a gap in implementing cutting-edge genetic tests, coupled with variation in diagnostic approaches among institutions. Therefore, rapid genetic diagnosis is increasingly guiding clinical decision-making and benefiting from targeted treatments [34,35]. The differential diagnosis of hypotonia in newborns includes muscular diseases such as infantile spinal muscular atrophy and congenital myopathies. For the study of these diseases, the performance of electromyography and muscle biopsy, which are invasive tests and may yield inconclusive results, is recommended.

Our findings, applicable to diagnosing patients with rare diseases, highlight challenges, including overlapping phenotypes, the risk of recurrence, the need for less invasive testing, and evaluating exam costs in the public health system. Changes in methylation profiles have implications for the emergence of various diseases, and studying these modifications can serve as biomarkers for prognostic purposes [36].

In our study, we specifically addressed the determination of the quality and quantity of DNA extracted from oral swab samples using NaCl procedures. These results were then compared with extractions performed using a commercial kit. Subsequently, the obtained material underwent MS–HRM analysis for loci associated with imprinting disorders, such as Prader–Willi and Angelman syndromes. We refrained from comparisons involving peripheral blood due to the discrepancy in sample numbers (2 × 110 swabs versus 20 blood tubes).

Commercial DNA extraction kits are commonly preferred due to their established credibility. Cost becomes a vital concern when considering a screening technique within a universal healthcare system like Brazil’s Unified Health System (SUS). Even small expenses become significant in such systems due to the test volume. In this study, extraction using the commercial kit resulted in lower yield, slightly longer execution time, and a cost twice as high as extraction using NaCl. In addition to the issue of purchasing extraction kits, it is worth highlighting that it is easier and cheaper, for example, to send oral swab tubes than blood tubes.

The choice of DNA extraction method is crucial for methylation analysis. While commercial kits offer high-quality DNA with low toxicity, collecting cells from an oral swab provides a convenient alternative, especially for vulnerable populations. The yield from extractions with NaCl is satisfactory for subsequent exome analyses, based on quantification by Qubit (all samples yielded equal to or greater than 100 ng in 30 μL) [37].

Saliva and buccal samples are increasingly utilized in medical research, including modern “omics” platforms. The oral cavity is an excellent source of biological material for genetic and comprehensive studies such as “omics” technologies [38].

Using swab-extracted material for whole genome sequencing (WGS) testing in genetic diagnosis is particularly interesting. By mapping genomic data, Kumar et al. (2023) identified the presence of unmapped reads in the human hg38 reference assembly. Analysis of these reads revealed the presence of microbial DNA. Upon analyzing the buccal swab and saliva samples, the level of microbial contamination did not significantly impact the diagnostic yield. Despite the common occurrence of bacterial and viral species in samples collected from the oral cavity, the diagnostic yield remains unaffected [39].

The purity and integrity of the DNA remain high, enabling further molecular analyses, such as Sanger sequencing. The next step involves using this material, extracted with NaCl, as a template for Next-Generation sequencing.

## 4. Conclusions

The comparison of extraction procedures shows that the simple NaCl extraction method is suitable for extracting DNA from a buccal swab sample. Successful sample collection and genomic DNA extraction from buccal swabs are noninvasive and reliable alternatives to invasive and uncomfortable blood collection for subjects and sample collectors. They are easier to mail from remote locations to diagnostic reference centers. Based solely on clinical examination, patients with imprinting defects are often difficult to diagnose in neonates and young children, requiring molecular analysis for a definitive diagnosis. The performance of a molecular test allowing rapid and accurate diagnosis is crucial for better prognosis in patients with rare diseases associated with imprinting defects. Additionally, we have demonstrated a simple method of sample collection and DNA extraction that provides a sufficient quantity and quality of DNA for PCR, qPCR, and Sanger DNA sequencing.

In summary, we use a technique for DNA extraction from oral swab cells using NaCl, which, compared to the commercial kit, showed lower cost and incredible speed. These findings suggest that this approach represents a reliable and more affordable method for obtaining DNA for genetic studies, particularly for methylation analysis.

## Data Availability

The datasets generated and analyzed during the current study are not publicly available due to patient data confidentiality and ethical aspects but are available from the corresponding author upon reasonable request.

## Supplementary Materials

The following supporting information can be downloaded at: https://www.mdpi.com/article/10.3390/genes15050641/s1, File S1: The primers were designed to bind only to the converted DNA (treated withbisulfite).; File S2: DNA concentration data, 260/280 and 260/230 absorbance ratios, and CT values from Commercial Kit and NaCl extractions, and CT values from RNASE P and SNRPN-SNURF genes amplifications; File S3: 0.8% agarose gel with products extracted by the different protocols

## Abbreviations

*AS*	Angelman syndrome
*BWS*	Beckwith–Wiedemann syndrome
*KOS*	Kagami–Ogata syndrome
*IFF*	Fernandes Figueira Institute
*MS–HRM*	Methylation-Sensitive High-Resolution Melting
*PHP1A*	pseudohypoparathyroidism type 1A
*PHP1B*	Pseudohypoparathyroidism type 1B
*PWS*	Prader–Willi syndrome
*SRS*	Silver–Russell syndrome
*TNDM*	transient neonatal diabetes mellitus type 1
*TS*	Temple syndrome
*UPD(14)mat*	Maternal uniparental disomy of chromosome 14
*UPD(14)pat*	Paternal uniparental disomy of chromosome 14
