# Supplementary material for: Establishing a Standardized DNA Extraction Method Using NaCl from Oral Mucosa Cells for Its Application in Imprinting Diseases Such as Prader–Willi and Angelman Syndromes: A Preliminary Investigation
Source: Genes (Basel). 2024 May 18;15(5):641. doi: 10.3390/genes15050641 (PMC11120874; doi:10.3390/genes15050641)
Supplement: Supplementary file 1 [file genes-15-00641-s001.zip › genes-2992304-supplementary.pdf]

Additional File S1

The primers were designed to bind only to the converted DNA (treated with bisulfite). Without treatment, there is no way for primers to attach to the region. As discussed in Ferreira et al (2019), both primers can bind to both alleles (see fusion curves) and act as a positive control for bisulfite conversion, a process due to the particularity of annealing in the treated DNA. The reverse primer shows perfect annealing in the two alleles. The forward primer presents a single incompatibility in the non-methylated allele (exchange of T for C) that does not interfere in the amplification capacity since there is still the pairing of ten nucleotides after this incompatibility.

Below is the sequence of the genomic region (sequences ID NG_012958.1 and NG_002690.1) containing the *SNRPN* and ***SNURF*** genes. The CpG islands are in bold type. Wild type: untreated bisulfite DNA

Wild-Type

tcattccggt gagggaggga gctgggaccc ctgcactgcg gcaaacaagc acgcctgcgc ggccgcagag gcaggctggc gcgcatgctc aggcggggat gtgtgcgaag cctgccgctg ctgcagcgag tctggcgcag agtggagcgg ccgccggaga tgcctgacgc atctgtctga ggagcggtca gtgacgcgat ggagcgggca aggtcagctg tgccggtggc ttctctcaag agacagcctg gggagcggcc acttttattc atcatcattc cggtgaggga gggagctggg accc

After DNA conversion, the non-methylated cytosines are converted in thymines, in both alleles. This condition allows the primers to be paired (place indicated by gray rectangles). The color red indicates the mismatch of the primer forward, in the non-methylated allele. For this reason, the presence of amplification is an indication that the DNA conversion worked correctly.

T indicates non-methylated cytosines converted to thymines.

Primers Sequence 5′-GGATTTTGTATTGCGGTAAATAAG-3′ and 5′-CAACTAACCTTACCCACTCCATC-3′

Non-methylated allele

tTattTTggt gagggaggga gTtgggaTTT TtgTaTtgTg gTaaaTaagT aTgTTtgTgT ggTTgTagag gTaggTtggT gTgTatgTtT aggTggggat gtgtgTgaag TTtgTTgTtg TtgTagTgag tTtggTgTag agtggagTgg TTgTTggaga tgTTtgaTgT atTtgtTtga ggagTggtTa gtgaTgTgat ggagTgggTa aggtTagTtg tgTTggtggT ttTtTtTaag agaTagTTtg gggagTggTT aTttttattT atTa

Methylated allele

tTattTcggt gagggaggga gTtgggaTTT TtgTaTtgcg gTaaaTaagT acgTTtgcgc ggTcgTagag gTaggTtggc gcgTatgTtT aggcggggat gtgtgcgaag TTtgTcgTtg TtgTagcgag tTtggcgTag agtggagcgg TcgTcggaga tgTTtgacgT atTtgtTtga ggagcggtTa gtgacgcgat ggagcgggTa aggtTagTtg tgTcggtggT ttTtTtTaag agaTagTTtg gggagcggTT aTttttattT atTa

Additional File S2

Table S1. DNA concentration data, 260/280 and 260/230 absorbance ratios, and CT values from Commercial Kit and NaCl extractions, and CT values from *RNASE P* and *SNRPN-SNURF* genes amplifications.

	[DNA] Nandrop		[DNA] Qubit		ABS 260/280		ABS 260/230		CT *SNRPN-SNURF*		CT *RNASE P*	
Patients	CK	NaCl	CK	NaCl	CK	NaCl	CK	NaCl	CK	NaCl	CK	NaCl
1	12,1	314,4	4,01	39,3	1,74	1,64	1,95	1,8	29,18	31,25205	24,43	27,16205
2	24,9	287,3	8,3	35,91	1,95	1,64	1,99	1,7	29,26	31,62	24,51	27,53
3	16,6	213,2	5,51	26,65	1,56	1,73	2	2,2	29,45	31,25205	24,71	27,16205
4	20,2	207,2	6,62	25,9	1,69	1,74	2,1	1,7	29,23	30,95671	24,48	26,86671
5	15	174,4	5,09	21,8	1,84	1,75	2	2,1	29,24	31,30467	24,5	27,21467
6	13,1	237,4	4,36	29,68	1,72	1,84	2	1,6	29,34	30,30467	24,6	26,21
7	23	232,5	7,62	29,06	1,89	1,67	2	2,5	27,54	29,61429	22,79	25,52
8	25,6	223,1	8,52	27,89	1,85	1,69	1,97	1,69	27,44	29,35	22,69	25,26
9	26,6	202,7	8,78	25,34	1,95	1,6	1,99	1,64	27,14	28,61429	22,39	24,52
10	24,6	234,8	2,18	29,35	1,82	1,69	1,98	1,65	27,689	29,25779	22,94	25,17
11	10,6	252,07	2,65	31,51	1,72	1,64	2,01	1,61	27,18779	29,08776	22,44	25
12	19	19,15	6,45	2,39	1,44	1,64	2	1,6	27,87786	29,75779	23,13	25,67
13	30,7	35,49	10,35	4,44	1,82	1,77	2	1,6	27,94402	28,9644	23,19	24,87
14	17,4	15,96	5,8	2,00	1,84	1,74	2,03	1,63	27,3944	28,6494	22,64	24,56
15	17,2	80,6	5,76	10,08	1,94	1,73	2,01	1,69	27,5944	29,95632	22,8444	25,87
16	45	176,47	14,87	22,06	2,02	1,83	1,99	1,61	29,25563	32,97609	24,50563	28,89
17	18	220,11	3,25	27,51	1,75	1,67	1,98	1,64	29,61256	33,50586	24,86256	29,42
18	12	125,04	3,99	15,63	1,73	1,69	1,99	1,65	30,08377	33,51687	25,33377	29,43
19	40,2	177,19	13,4	22,15	1,93	1,61	1,99	1,68	30,04425	34,54925	25,29425	30,46
20	26,5	30,59	8,74	3,82	1,92	1,69	1,97	1,67	30,13923	35,83009	25,38923	31,74
21	27,3	133,43	9,02	16,68	1,8	1,74	1,99	1,69	30,75756	35,92466	26,00756	31,83
22	27,1	151,14	8,74	18,89	1,92	1,78	1,92	1,5	28,13987	33,24836	23,38987	29,16
23	16,8	192,66	5,6	24,08	1,84	1,44	1,94	1,59	29,85644	33,38955	25,10644	29,3
24	16,9	213,95	5,45	26,74	2,05	1,58	1,98	1,56	29,90956	33,41968	25,15956	29,33
25	16,1	156,1	5,32	19,51	2,03	1,45	1,97	1,58	28,87419	32,86865	24,12419	28,78
26	17,8	29,78	5,84	3,72	1,84	1,54	1,97	1,57	29,09006	32,87124	24,34006	28,78
27	16	8,24	5,23	1,03	2,05	1,57	1,99	1,61	29,35381	33,01922	24,60381	28,93
28	16	3,82	5,33	0,48	1,85	1,62	1,96	1,62	28,74609	33,51763	23,99609	29,43
29	40,5	34,91	13,5	4,36	1,9	1,45	1,95	1,65	29,32835	33,76423	24,57835	29,67
30	16,6	138,64	5,41	17,33	2,1	1,47	1,97	1,67	29,53071	33,96737	24,78071	29,87737
31	39,1	51,44	12,98	6,43	1,98	1,87	1,99	1,69	30,02756	32,45756	25,27756	28,36756
32	13,3	71,85	4,25	8,58	1,9	1,77	1,99	1,8	27,40987	29,12987	22,65987	25,03987
33	13,9	32	4,23	4	2,02	1,55	1,98	1,8	27,52644	29,24644	22,77644	25,15644
34	18,2	164,83	5,91	20,14	1,69	1,66	1,99	1,6	29,17956	30,89956	24,42956	26,80956
35	16,2	86,57	5,4	10,52	1,79	1,88	1,97	2,1	28,14419	29,86419	23,39419	25,77419
36	18,2	14,62	5,87	2,87	2,2	1,87	1,99	1,69	28,36006	30,08006	23,61006	25,99006
37	8,8	72,92	6,12	9,11	2,17	1,85	1,98	1,61	28,62381	30,34381	23,87381	26,25381
38	10,1	95,1	3,45	11,25	2,04	1,75	2,01	1,64	28,01609	29,73609	23,26609	25,64609
39	20,9	47,87	6,45	5,85	1,91	1,84	2	1,66	28,59835	30,31835	23,84835	26,22835
40	24,6	199,42	8,2	24,52	2,03	1,67	1,99	1,63	28,80071	30,52071	24,05071	26,43071

41	16,7	62,78	5,33	7,84	2,02	1,69	1,99	1,65	28,87022	30,59022	24,12022	26,50022
42	15,9	23,61	5,3	2,95	2,01	1,6	1,94	1,66	29,40923	31,12923	24,65923	27,03923
43	26,8	32,78	8,63	4,10	1,95	1,69	1,99	1,68	27,52644	29,24644	22,77644	25,15644
44	14,3	113,89	4,69	14,24	1,88	1,64	1,98	1,64	28,36006	30,08006	23,61006	25,99006
45	11,7	97,69	3,87	12,21	1,91	1,64	1,99	1,64	28,59835	30,31835	23,84835	26,22835
46	10,1	47,27	3,56	5,91	2,21	1,77	2,01	1,61	28,52563	30,24563	23,77563	26,15563
47	15,7	290,07	5,23	36,26	1,97	1,74	2	1,61	28,01609	29,73609	23,26609	25,64609
48	12,7	265,07	4,66	33,13	1,82	1,73	1,97	1,62	28,01609	29,73609	23,26609	25,64609
49	35,9	191,77	10,55	23,97	2,12	1,74	1,97	1,63	28,14419	29,86419	23,39419	25,77419
50	29,6	178,07	9,48	22,26	2,02	1,87	1,95	1,65	27,40987	29,12987	22,65987	25,03987
51	43,4	148,36	14,26	18,55	1,85	1,77	1,96	1,66	28,18563	29,90563	23,43563	25,81563
52	58,2	212,09	19,4	26,51	1,9	1,55	1,99	1,62	28,39126	30,11126	23,64126	26,02126
53	55,3	207,59	18,43	25,95	2	1,66	2,02	1,62	29,01377	30,73377	24,26377	26,64377
54	49,2	203,39	16,4	25,42	2	1,88	2,08	1,68	28,97425	30,69425	24,22425	26,60425
55	60,3	197,69	20,1	24,71	2	1,87	2,01	1,61	29,06923	30,78923	24,31923	26,69923
56	40,3	211,31	13,43	26,41	2,01	1,85	2	1,6	29,68756	31,40756	24,93756	27,31756
57	41	182,67	13,67	22,83	2	1,75	2	1,6	27,06987	29,78987	22,31987	25,69987
58	44,5	140,67	14,83	17,58	1,99	1,84	2,01	1,66	27,18644	28,90644	22,43644	24,81644
59	25,1	198,27	8,37	24,78	1,96	1,67	1,99	1,79	28,83956	31,15956	24,08956	27,06956
60	20,1	199,17	6,7	24,90	1,91	1,7	1,98	1,71	27,80419	29,52419	23,05419	25,43419
61	19,7	93,87	6,57	11,73	2,22	1,71	1,98	1,75	28,02006	29,74006	23,27006	25,65006
62	19,1	127,77	6,37	15,97	2,05	1,74	1,99	1,71	28,28381	30,08006	23,53381	25,99006
63	15,2	425,87	5,07	53,23	1,92	1,73	1,99	1,7	27,67609	29,39609	22,92609	25,30609
64	13,8	277,87	4,6	34,73	1,87	1,72	1,99	1,7	28,25835	29,97835	23,50835	25,88835
65	16,8	287,47	5,6	35,93	1,96	1,82	1,99	1,7	28,46071	31,18071	23,71071	27,09071
66	23,2	210,87	7,73	26,36	2,12	1,54	1,98	1,7	28,53022	30,25022	23,78022	26,16022
67	16,3	255,67	5,43	31,96	2,18	1,53	1,95	1,7	29,06923	30,78923	24,31923	26,69923
68	15,3	22,75	5,1	2,84	2,01	1,52	1,97	1,7	27,58644	29,50644	22,83644	25,41644
69	23,2	39,09	7,73	4,89	1,97	1,84	1,99	1,63	28,42006	30,34006	23,67006	26,25006
70	45,4	19,56	15,13	2,45	1,84	1,72	1,99	1,65	28,65835	31,57835	23,90835	27,48835
71	51	84,2	17	10,53	2,1	1,46	1,99	1,66	28,58563	30,50563	23,83563	26,41563
72	55	180,07	18,33	22,51	2	1,63	2	1,62	28,01761	30,08609	23,26761	25,99609
73	27,4	223,71	9,13	27,96	1,95	1,65	1,98	1,64	28,07609	30,00999	23,32609	25,91999
74	38,7	128,64	12,90	16,08	1,82	1,67	1,99	1,69	28,20419	30,12419	23,45419	26,03419
75	20,4	180,79	6,80	22,60	1,45	1,64	1,98	1,68	27,46987	29,38987	22,71987	25,29987
76	19,9	34,19	6,63	4,27	1,94	1,66	1,98	1,61	28,85563	30,77563	24,10563	26,68563
77	17,8	137,03	5,93	17,13	1,99	1,78	1,94	2,1	29,06126	30,98126	24,31126	26,89126
78	34,2	154,74	11,40	19,34	1,78	1,77	2	1,6	29,68377	31,60377	24,93377	27,51377
79	44,7	196,26	14,90	24,53	1,83	1,63	2,12	1,72	29,64425	32,56425	24,89425	28,47425
80	17	217,55	5,67	27,19	1,89	1,84	2,09	1,69	29,73923	32,65923	24,98923	28,56923
81	56,5	159,7	18,83	19,96	1,89	1,6	2,11	1,71	28,35756	32,27756	23,60756	28,18756
82	66	33,38	22,00	4,17	1,52	1,61	2,11	1,71	27,73987	30,65987	22,98987	26,56987
83	73,3	11,84	24,43	2,58	1,84	1,58	2,01	1,61	27,85644	30,77644	23,10644	26,68644
84	56,2	7,42	18,73	2,25	1,73	1,55	2	1,6	29,50956	31,42956	24,75956	27,33956
85	19,4	38,51	6,47	4,81	1,87	1,76	2	1,6	28,47419	30,39419	23,72419	26,30419
86	20,6	142,24	6,87	17,78	2,1	1,63	1,99	1,63	28,69006	30,61006	23,94006	26,52006

87	66,2	55,04	22,07	6,88	2,1	1,77	1,99	1,69	28,95381	31,87381	24,20381	27,78381
88	16,6	75,45	5,53	9,43	1,97	1,68	1,98	1,61	28,34609	30,26609	23,59609	26,17609
89	12,7	35,6	4,23	4,45	1,98	1,8	1,99	1,64	28,92835	31,14835	24,17835	27,05835
90	22,6	168,43	7,53	21,05	1,96	1,77	2	1,65	29,13071	31,05071	24,38071	26,96071
91	14,9	90,17	4,97	11,27	2	1,48	2,01	1,68	29,20022	31,12022	24,45022	27,03022
92	45,1	18,22	15,03	2,28	1,88	1,87	1,99	1,67	29,73923	31,65923	24,98923	27,56923
93	31,1	76,52	10,37	9,57	1,89	1,77	1,99	1,69	27,85644	31,09644	23,10644	27,00644
94	19,8	98,7	6,60	12,34	1,79	1,67	2,01	1,62	28,69006	32,61006	23,94006	28,52006
95	19,5	51,47	6,50	6,43	1,72	1,65	2,01	1,61	28,92835	31,84835	24,17835	27,75835
96	16,7	203,02	5,57	25,38	1,93	1,7	2	1,65	28,85563	32,77563	24,10563	28,68563
97	50,6	66,38	16,87	8,30	1,71	1,71	2	1,66	28,00101	30,26609	23,25101	26,17609
98	38,4	27,21	12,80	3,40	1,71	1,69	1,99	1,71	28,94609	31,06609	24,19609	26,97609
99	12,8	29,78	4,27	3,72	1,82	1,64	1,97	1,57	28,87419	30,79419	24,12419	26,70419
100	12,8	88,24	4,27	13,58	1,88	1,77	2,01	1,61	27,73987	30,19874	22,98987	26,10874
101	16,9	66,24	5,63	13,25	2,05	1,77	2,01	1,79	28,18563	31,12923	23,59609	26,17609
102	20,6	66,87	6,87	13,37	1,92	1,74	1,99	1,68	28,39126	29,24644	24,17835	27,05835
103	23,2	101,23	7,73	20,25	1,87	1,73	1,99	1,71	29,01377	30,08006	24,38071	26,96071
104	29,6	185,36	9,87	37,07	1,96	1,74	2,01	2,1	28,97425	30,31835	24,45022	27,03022
105	22,6	120,36	7,53	24,07	2,12	1,87	2,01	1,6	29,06923	30,24563	24,98923	26,46923
106	16,2	123,2	5,40	24,64	2,18	1,77	2	1,72	29,68756	29,73609	23,10644	26,89644
107	20,23	100,2	6,74	20,04	2,01	1,55	2	1,79	27,06987	29,73609	23,94006	27,25006
108	21,45	100,58	7,15	20,12	1,97	1,66	1,99	1,71	27,18644	29,86419	24,17835	26,75835
109	19,88	135,65	6,63	27,13	1,84	1,88	1,94	1,71	28,83956	29,12987	24,10563	27,45563
110	18,2	114,28	6,07	22,86	1,88	1,77	2,01	1,61	27,80419	29,90563	22,98987	26,10874
Media	26,654	132,5846	8,6093636	17,215938	1,9158182	1,698	1,9937273	1,6859091	28,594482	30,88053	23,873405	26,878476
Standard Deviation	15,10742	89,68191	4,8633436	10,796815	0,1460285	0,1074142	0,0320755	0,1353601	0,8039944	1,4770255	0,7845616	1,4434

Ck:Commercial Kit

Additional File S3

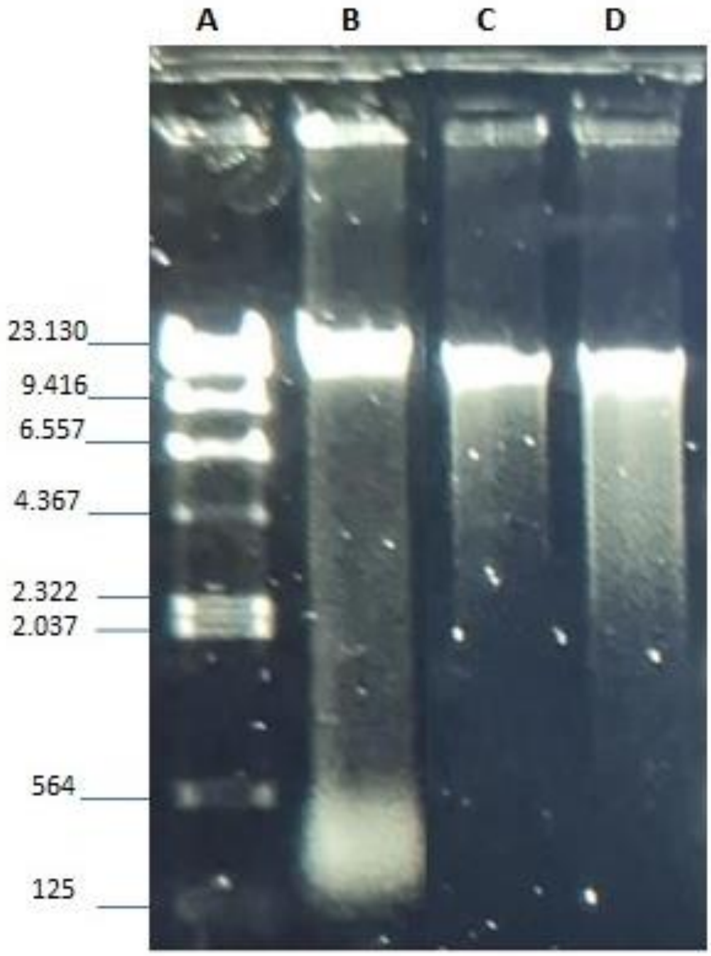

•

Electrophoresis in 0.8% agarose gel, stained with ethidium bromide (0.5 µg/ml), of genomic DNAs extracted with different methods. In A: Molecular weight marker (λDNA/Hind III fragments, Invitrogen), B: DNA extracted from oral swab by NaCl, C: DNA extracted from peripheral blood with the commercial kit; D: DNA extracted from oral swab with a commercial kit. The molecular mass marker (λDNA/Hind III fragments, Invitrogen) is expressed in bp.
